# Ontologies for the Description of Mouse Phenotypes

**DOI:** 10.1002/cfg.430

**Published:** 2004

**Authors:** G. V. Gkoutos, E. C. J. Green, A.-M. Mallon, A. Blake, S. Greenaway, J. M. Hancock, D. Davidson

**Affiliations:** 1 MRC Mammalian Genetics Unit Harwell Oxfordshire OX11 0RD UK; 2 MRC Human Genetics Unit Edinburgh UK

## Abstract

Ontologies are becoming increasingly important for the efficient storage, retrieval and mining of biological data. The description of phenotypes using ontologies is a particularly complex problem. We outline a schema that can be used to describe phenotypes by combining orthologous axiomatic ontologies. We also describe tools for storing, browsing and searching such complex ontologies. Central to this approach is that assays (protocols for measuring phenotypic characters) describe what has been measured as well as how this was done, allowing assays to link individual organisms to ontologies describing phenotypes. We have evaluated this approach by automatically annotating data on 600 000 mutant mice phenotypes using the SHIRPA protocol. We believe this approach will enable the flexible, extensible and detailed description of phenotypes from any organism.


**Comparative and Functional Genomics**
*Comp Funct Genom* 2004; **5**: 545–551.
Published online in Wiley InterScience (www.interscience.wiley.com). **DOI:** 10.1002/cfg.430

**Conference Paper**

### Ontologies for the description of mouse phenotypes

G. V. Gkoutos[1]*, E. C. J. Green[1], A.-M. Mallon[1], A. Blake[1], S. Greenaway[1], J. M. Hancock[1] and D. Davidson[2]
[1]*MRC Mammalian Genetics Unit, Harwell, UK*
[2]*MRC Human Genetics Unit, Edinburgh, UK*

**Correspondence to:*
*G. V. Gkoutos, MRC Mammalian Genetics Unit, Harwell, Oxfordshire, OX11 0RD, UK.*
*E-mail: g.gkoutos@har.mrc.ac.uk*

Received: 12 October 2004
Accepted: 18 October 2004

### Abstract

**Ontologies are becoming increasingly important for the efficient storage, retrieval and mining of biological data. The description of phenotypes using ontologies is a particularly complex problem. We outline a schema that can be used to describe phenotypes by combining orthologous axiomatic ontologies. We also describe tools for storing, browsing and searching such complex ontologies. Central to this approach is that assays (protocols for measuring phenotypic characters) describe what has been measured as well as how this was done, allowing assays to link individual organisms to ontologies describing phenotypes. We have evaluated this approach by automatically annotating data on 600 000 mutant mice phenotypes using the SHIRPA protocol. We believe this approach will enable the flexible, extensible and detailed description of phenotypes from any organism. Copyright © 2004 John Wiley & Sons, Ltd.**

**Keywords:** **phenotype ontology; phenotype description; PATO; bioinformatics**

### Introduction

### Mouse mutant phenotypes

The completion of the human genome sequence heralds a new era of understanding of the genetic basis of human disease. Determining the function of every one of the human genes and their role in disease will be greatly assisted by the development and characterization of mouse models of human disease. However, assessing the effect on the organism of any change made in a gene will require systematic screens and tests that allow us to describe the phenotypic consequences in a comprehensive way. An increasing number of laboratories and companies worldwide are now carrying out detailed analysis of mouse phenotypes that have been generated from the large-scale mutagenesis (Balling, 2001) of the mouse genome.

Large-scale projects, such as EUMORPHIA (**http://www.eumorphia.org**), in aiming at the standardization and dissemination of primary and secondary phenotyping protocols for all body systems in the mouse, produce consistent methodologies for systematic and standardized characterization of mouse phenotype. This requires managing information about mutants in a paperless environment, and building of databases that will allow this data to be shared between laboratories and used to formulate hypotheses about gene function. The key to satisfying this need is the ability to describe different phenotypes in a consistent and structured way.

### Bio-ontologies

Ontologies have their root in Greek philosophy and aim at the description of what exists. An ontology is a formal specification of entities and their relationships. In biology, since the advent of the Gene Ontology (GO) (Gene Ontology Consortium, 2000), ontologies have been used to specify the semantic relationships among terms, and have become a standard used to support knowledge representation in the field of genomics (GO Consortium, 2004). Bio-ontologies have been

Copyright © 2004 John Wiley & Sons, Ltd.

employed for scientific data integration and exploration, clarifying scientific exchange by providing a shared vocabulary, and extending the power of computational approaches and systems to perform data exploration, inference and mining (Blake, 2004).

### Phenotype ontologies

Phenotype information has traditionally been captured in a free-text manner. Free text searching also forms the basis of information mining and retrieval, but it is extremely limited because of an inherent lack of accuracy and specificity. Complex free text descriptions, such as are used for phenotypes, are almost impossible to index and retrieve in a useful way and yet advanced searches are required to fully exploit and realize the potential of these data. With the success of the use of bio-ontologies in addressing these problems in other areas of biology, it seems logical to use ontologies to describe phenotypes.

However, phenotypic descriptions present a major conceptual and practical problem that cannot be addressed by the relatively simplistic approach that has been used to describe the features of well-defined domains of most other bio-ontologies. Hence, in order to describe complex biological areas of knowledge, such as the description of mutant phenotypes, a more sophisticated methodology is required. The Mouse Genome Database (Blake, 2003) team have developed the Mammalian Phenotype Ontology (MPO: **http://obo.sourceforge.net/cgi-bin/detail.cgi?musphen**) to describe mouse phenotypes in a GO-like manner. This development takes a pragmatic approach whereby instances are added as needed to annotate mouse phenotypes. As the developers are part of the Phenotype Consortium (see below), the database has been created in such a way that it can easily be extended to form instances of the compositional approach used in the schema described here.

### A compositional approach for describing mouse phenotypes

The Phenotype Consortium, part of the GO consortium, was formed with the objective of addressing the issue of representing phenotype information. The consortium decided that a minimal set of knowledge domains or core ontologies should be part of forming phenotype ontologies. These domains are:

- Anatomy
- Ontogeny (developmental anatomy)
- Behaviour
- Pathology
- Cell types
- GO

Furthermore, and perhaps more importantly, the need for a methodology for expressing phenotype information that could be extended to any organism, achieving interoperability between individual species communities, was highlighted.

### Phenotype and trait ontology (PATO)

To that effect, Ashburner proposed the Phenotype and Trait Ontology (PATO) (available at the Open Biological Ontologies (OBO) site: **http://obo.sourceforge.net/**) with the objective of capturing information about phenotypes in any organism. The idea behind this proposal is that PATO would form a common platform upon which different ontologies (such as behaviour, anatomy, etc.) would be mapped to provide a consistent representation of phenotypic data. PATO would provide for concepts (deriving from different core ontologies) a set of relative attributes, their corresponding values and the assays that were employed to define these. The combination of the concepts, attributes, values and assays would form the basis for the systematic description of phenotype.

Figure 1 presents a schema for phenotype representation based on this concept (Gkoutos *et al.*, 2004). According to this schema, the whole organism has certain attributes, as presented in Table 1, and exists under certain handling conditions.

The organism also has a set of core components: its anatomy, development, physiology and behaviour. Each of these core components is represented by a separate ontology and each has a set of attributes, again represented by an ontology. For example, the organism may have an anatomical component *left eye*, which is a term from the anatomy ontology. The left eye, in turn, may have attributes of *colour, size*, etc., taken from the attributes ontology. This combination of core concept and attribute constitutes a phenotypic character — something that can be measured. Phenotypic

Copyright © 2004 John Wiley & Sons, Ltd. 

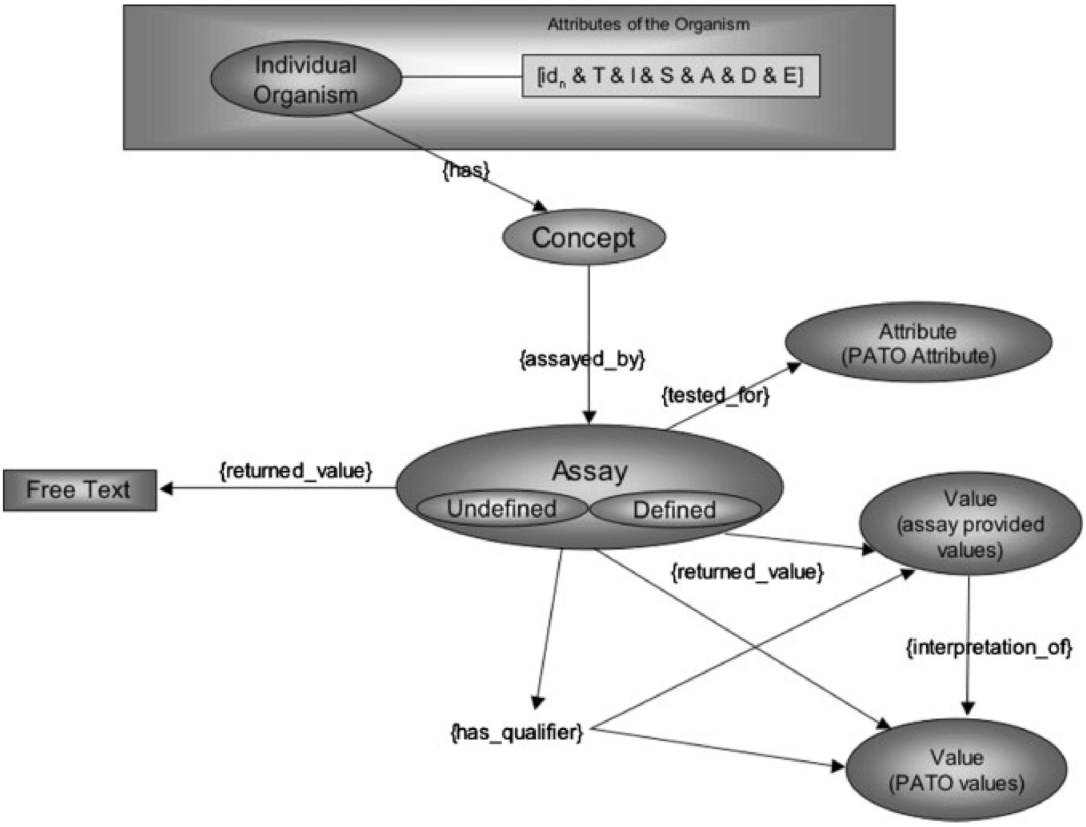

**Figure 1.** Proposed schema by Ashburner, Davidson and Gkoutos (modified from Gkoutos *et al.*, 2004). Organism attributes: id, identifier for individual (*n*); T, species; G, genotype (I, strain; S, genotypic sex; A, alleles at named loci); E, handling conditions; D, age/stage of development

**Table 1.** Organism attributes

| id | Identifier for individual (*n*) |
|---|---|
| T | Species (e.g. NCBI taxonomy browser; **http://www.ncbi.nlm.nih.gov/Taxonomy/**) |
| G | Genotype |
| | I Strain (e.g. MGI; **http://www.informatics.jax.org/**) |
| | S Genotypic sex |
| | A Alleles at named loci (e.g. MGI; **http://www.informatics.jax.org/**) |
| E | Handling conditions (i.e. EUMORPHIA) |
| D | Age/stage of development (Theiler, 1989; and other staging criteria, e.g. EMAP; **http://genex.hgu.mrc.ac.uk/Databases/Anatomy/MAstaging.html**) |

characters, in turn, link to an ontology of *assays* which return a variety of *values*, again represented by an ontology.

### How it will work

When this schema is used to describe actual phenotypes, instances of single phenotypic characters are linked together to provide a full phenotypic description of an individual organism. Each character can be represented by a line in a table, where the table represents the full phenotype (Figure 2). In other words, phenotypic character instances and associated phenotypic instances are only linked in the knowledgebase (an ontology together with a set of individual instances of the kinds of entities it specifies; Stevens, 2004).

### Storing and accessing phenotype ontologies

### Database

A database was designed to reflect the functionality of the schema proposed for modelling phenotype ontologies. The relational schema is based on

Copyright © 2004 John Wiley & Sons, Ltd. 

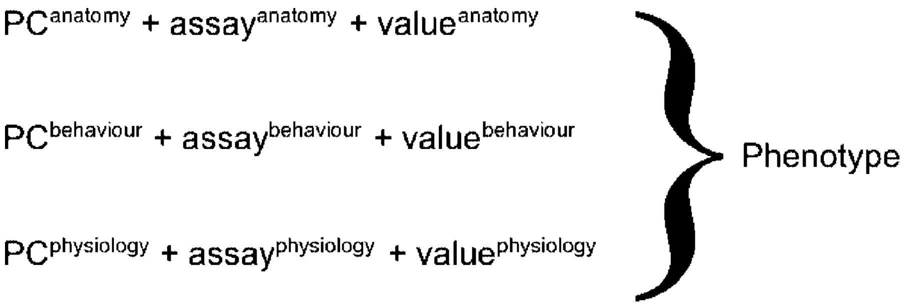

**Figure 2.** Schematic of phenotype description as the sum of the results of assaying different characters. PC, phenotypic character the functionality of Gene Ontology database but allows the storage of multiple phenotype ontologies of different species and domains. Furthermore, the database is designed to hold instances of phenotypic descriptions, generating knowledgebases for individual domains and allowing cross-referencing and indexing between them. The relational schema was implemented in a MySQL (**http://www.mysql.com/**) database and the SQL code for building and populating the database can be obtained from the authors.

We have also written parsers in Perl that take ontologies in OBOL format (**http://obo.sourceforge.net/**), parse them into SQL statements, and load them into the databases. We are currently in the process of generating parsers that would automatically load ontologies expressed in DAML+OIL/OWL into the database.

Due to the nature of our schema and the constant community input required for the success of this proposal, a mechanism was required that could easily access, update and reflect any change to our schema proposal. For this purpose, we have created a set of tools, collectively known as Chameleon.

### Chameleon

Database designs are rarely stable and are instead likely to change quite frequently, depending on new features required from the system. Using our schema approach, this would require quite significant re-coding of any applications each time a change is made. In order to minimize code maintenance and initial development times, a multilayer model was developed (Figure 3).

The lowest 'database' layer allows the data contained in the database to be accessed as Java objects and also maintain these objects in server memory, allowing very fast data manipulation. A 'middle' layer provides higher functions, such as searching, and this layer provides an API to application programs.

Chameleon is a program that automatically generates source code that implements the low database layer in a matter of seconds. Each database table becomes a Java object class with methods to allow fetching, editing, creating and deleting data via a Java object. The classes written and the methods they contain are dependent on the structure of the database table. Database design changes now result in a small number of middleware code changes, rather than changing application code.

Chameleon can be set to produce code that can be read only or read/write. Any type of SQL database can be supported, so long as there is a suitable JDBC (**http://java.sun.com/j2se/1.5.0/docs/api/**) driver that supports SQL meta-data. When the resultant Java classes are first used, all the data from the database is read into memory and indexes are built. The JVM memory size (**http://java.sun.com/j2se/1.5.0/docs/tooldocs/windows/java.html**) may need to be adjusted to enable this. This reading of data will lead to a small delay the first time the classes are accessed. When used under an application, such as JRUN or Tomcat, the data will be loaded once for all users of the database classes. Any changes to the data objects will thus be instantly reflected in other applications data objects (in the read/write model). An API method call can also be used to force the classes to reload themselves. Applications can also register a call-back interface to enable them to be informed if the underlying data has changed.

Chameleon creates the object-relational mapping as 'personalized' Java source code, which

Copyright © 2004 John Wiley & Sons, Ltd. 

is then compiled as part of the application's code base. Unlike a system such as Hibernate (**http://www.hibernate.org/**), which acts as an active layer between the application and the database, it is a 'run once and forget' system. Using Chameleon, the mapping layer can be distributed simply as code or within a jar/war file without requiring local installation of additional software.

Although Chameleon itself does not produce code to manipulate hierarchical data, it provides a simple and efficient way of implementing it. Chameleon is written in Java 1.5.0 beta compliant code and will not compile under other versions of Java, although it will run under Java 1.4 JREs.

We have employed Chameleon to allow us to access our test-bed ontology and produced a visualization tool, termed CRAVE.

### Visualizing and searching phenotype ontologies

### Concept Relation Assay Value Explorer (CRAVE)

CRAVE was developed due to the need to be able to navigate and visualize complex ontologies, such as those developed based on the proposed schema, that the current bio-ontologies browsers available on the Gene Ontology Consortium Website (**http://www.geneontology.org/**) could not represent. It is a visualization tool that accesses these ontologies using the Chameleon package described above. It is based on a variety of open-source Java classes and developer tools, plus our own custom software engineering. We chose Java and JavaScript to achieve platform independence.

Figure 4 shows the browser interface. CRAVE is open source and can be accessed at: **http://www.mgu.har.mrc.ac.uk/servlet/browser.frameset**

### Searching the ontologies

CRAVE performs Boolean and advanced searches in a simple, single interface. So, besides the Boolean 'AND', 'OR' and 'NOT' operations, it allows organism and domain-specific searches. Hence the user, or an external application, can query the ontology based on organism, specific domains, or even on individual groups of ontologies. It employs 'one-line commands' to facilitate reverse engineering of the API by external applications.

CRAVE queries the ontologies in a case-insensitive manner, converting any punctuation mark (i.e. underscore or slash character, etc.) used in the term name to white space and allow users to search for them either way, e.g. *body_position* could be searched for as *body_position* or as *body position*, or even *BODY POSITION*.

Finally, although ontologies should hold synonyms for term names and different spellings (e.g. British vs. American English), we have adopted, in a separate standalone database, lists of terms spelled differently according to British and American spelling and are populating common synonyms to allow CRAVE to search for them.

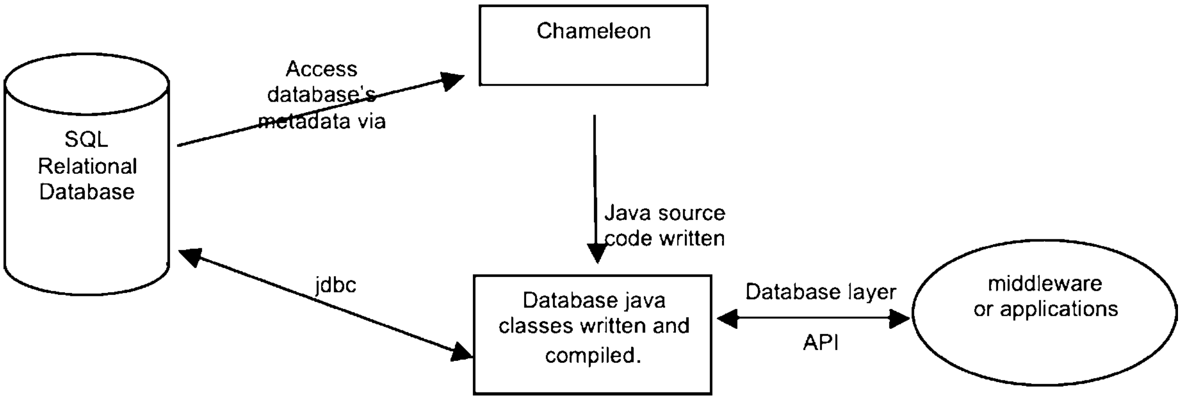

**Figure 3.** Schema showing Chameleon

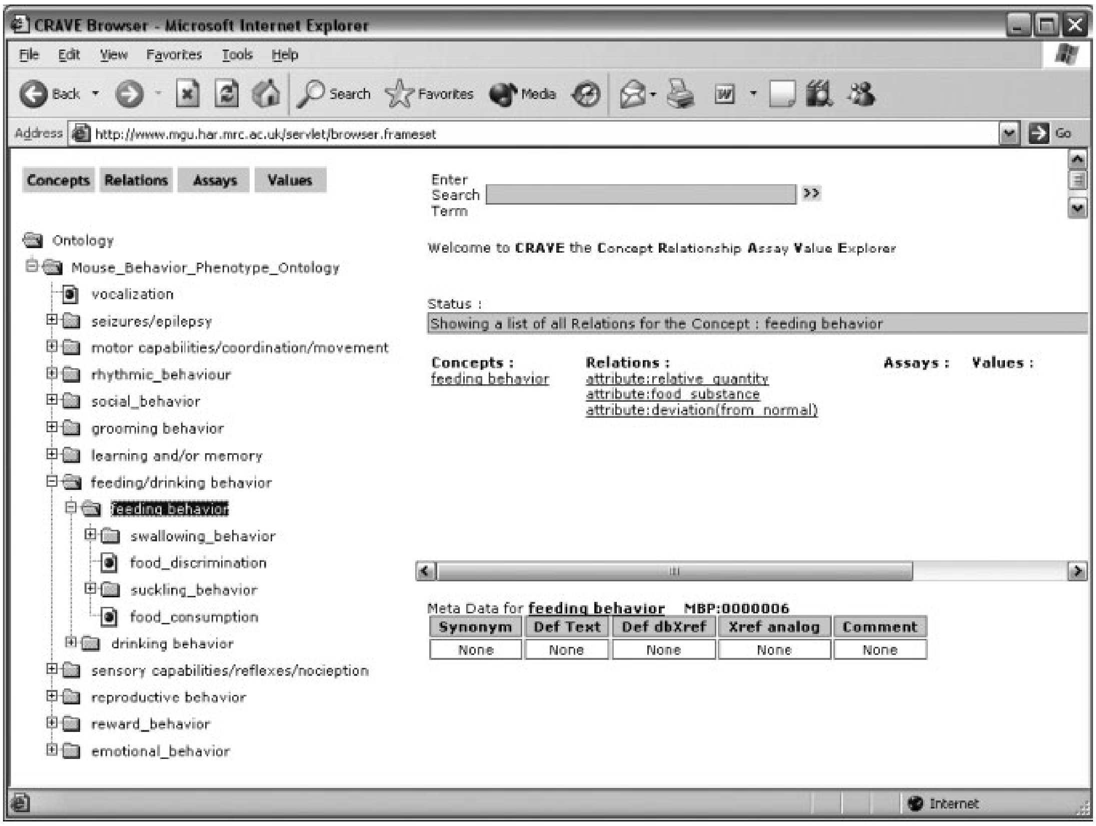

**Figure 4.** The CRAVE interface

### Applying phenotype ontologies

### Populating the assay ontology and automating phenotype annotation

Assays are central to the schema in Figure 1. EUMORPHIA is producing agreed, validated standarized operating procedures (SOPs) for mouse phenotyping. These are in many ways equivalent to those assays. We have converted the SOPs produced by EUMORPHIA into XML format, placed them in a database and they are currently being validated by the EUMORPHIA community. These SOPs will be accessed online by a browser produced by us available at: **http://www.eumorphia.org/ECFLP** and will be added to the Assay ontology, allowing us to automatically annotate phenotypes based on associations between assays and the phenotypic characters they measure. As a trial, we have employed our test-bed ontology, the mouse behaviour ontology, and have used this approach to annotate around 600 000 mice that were tested for different aspects of behaviour using a collection of behavioural assays, collectively known as the SHIRPA protocol (Rogers *et al.*, 2001). The test-bed ontology was based on the Mammalian phenotype ontology (Blake, 2003), which was purposely designed in such a way that it could be easily mapped into our schema proposal. The assays that were employed to characterize the behaviour of these mice were added to the assay ontology as part of the PATO ontology and the annotations were automatically generated via the Chameleon packages, described above. Having successfully annotated the recorded phenotypes we plan to apply some basic statistical analysis to answer questions such as whether we can automatically detect any dependencies between phenotype observations.

### Conclusions

The schema we have proposed allows extensibility and interoperability. Although ontology should

Copyright © 2004 John Wiley & Sons, Ltd. 

not cover all possible information about a domain, the main idea behind this concept is to allow phenotype ontologies to cope with novel and unpredictable phenotypes and account for new assays, serving scientific autonomy and information validity and integrity. We have created methodologies and databases that allow uploading and storing of ontologies modelled based on this schema, and dynamic update of different parts of the core ontologies, including PATO, without the loss of applied facets. We have also implemented a browser which allows searching and viewing of the knowledge captured though the PATO relations.

The approach described here has been discussed at a theoretical level at a number of international workshops, including the Phenotype Consortium meeting held in Bar Harbor in September 2003, as well as with representatives of the Mouse Genome Database and Mouse Phenome Project database (**http://www.jax.org/phenome**), the major international repositories for phenotype data in the mouse. As an example, we plan to use it to annotate data emerging from EUMORPHIA, thereby demonstrating the utility of the approach and allowing it to be implemented in other relevant databases.

### Acknowledgements

This project is funded by the European Commission under contract number QLG2-CT-2002-00930. We thank Michael Ashburner and Suzie Lewis for their comments and Pat Nolan and Valter Tucci for discussions on mouse behaviour.

### References

Balling R. 2001. ENU mutagenesis: analyzing gene function in mice. *Annu Rev Genomics Hum Genet* **2**: 463–492.

Blake J. 2004. Bio-ontologies — fast and furious. *Nature Biotechnol* **22**: 773–774.

Blake JA, Richardson JE, Bult CJ, Kadin JA, Eppig JT and the members of the Mouse Genome Database Group. 2003. MGD: the Mouse Genome Database. *Nucleic Acids Res* **31**: 193–195.

EMAP staging criteria: **http://genex.hgu.mrc.ac.uk/Databases/Anatomy/MAstaging.html**

EUMORPHIA: **http://www.eumorphia.org**

Gene Ontology Consortium Website: **http://www.geneontology.org/**

Gkoutos GV, Green ECJ, Mallon A, Hancock JM, Davidson D. 2004. Building mouse phenotype ontologies. *Pac Symp Biocomput* **9**: 179–189.

GO Consortium. 2004. The Gene Ontology (GO) database and informatics resource. *Nucleic Acids Res* **32**: 258–261.

Mammalian Phenotype Ontology (MPO): **http://obo.sourceforge.net/cgi-bin/detail.cgi?musphen**

Mouse Genome Informatics (MGI): **http://www.informatics.jax.org/**

MySQL: **http://www.mysql.com/**

NCBI taxonomy: **http://www.ncbi.nlm.nih.gov/Taxonomy/**

Open Global Ontologies, OBO: **http://obo.sourceforge.net/**

Mouse Phenome Project: **http://www.jax.org/phenome**

Rogers DC, Peters J, Martin JE, *et al*. 2001. *Neurosci Lett* **306**(1–2): 89–92.

Stevens R. 2004. Knowledge base. In *Dictionary of Bioinformatics and Computational Biology*, Hancock JM, Zvelebil MJ (eds). Wiley: New York.

Sun Microsystems. Java 1.5.0 API: **http://java.sun.com/j2se/1.5.0/docs/api/**

Sun Microsystems. Java VM Runtime: **http://java.sun.com/j2se/1.5.0/docs/tooldocs/windows/java.html**

The Gene Ontology Consortium. 2000. Gene Ontology: tool for the unification of biology. *Nature Genet* **25**: 25–29.

Theiler K. 1989. *The House Mouse: Atlas of Embryonic Development*. Springer: New York.

Copyright © 2004 John Wiley & Sons, Ltd.
